# Probiotics ingestion prevents HDAC11-induced DEC205+ dendritic cell dysfunction in night shift nurses

**DOI:** 10.1038/s41598-019-54558-4

**Published:** 2019-11-29

**Authors:** Hui Yang, Jing Yang, Hui Cheng, Huili Cao, Shan Tang, Qiaohong Wang, Juan Zhao, Baohua Li, Yongxia Ding, Chang Ma

**Affiliations:** 1 Department of Nursing, First Hospital of Shanxi Medical University, Taiyuan, China; 2 Department of Respirology, Second Affiliated Hospital, Harbin Medical University, Harbin, China

**Keywords:** Risk factors, Lymphocytes

## Abstract

It is known that the day-night shift-rotation has a negative impact on the immune system. The underlying mechanism remains to be further investigated. Probiotics have regulatory effects on immune functions. This study aims to investigate the role of probiotic ingestion in preventing the DEC205^+^ dendritic cell (decDC) dysfunction in day-night shift-engaging nurses. In this study, blood samples were collected from day-night shift-rotating nurses who took or did not take yogurt (containing *C. Butyricum*) during the night shift (NS). decDC functions were evaluated with pertinent immunological approaches. We observed that the immune tolerogenic functions and interleukin (IL)-10 expression were impaired in decDCs of nurses after NS. HDAC11 was detected in decDCs that was markedly up regulated after NS. The HDAC11 levels were negatively correlated with the immune tolerogenic functions in decDCs. Ingestion of probiotic-containing yogurt during NS efficiently suppressed Bmal1 and HDAC11 levels as well as up regulated the immune regulatory functions in decDCs. In conclusion, NS has a negative impact on decDC immune tolerogenic functions, which can be prevented by ingesting probiotics-containing yogurt during NS.

## Introduction

Circadian rhythm disruptions occur in daily life frequently, such as day-night shift rotation and jet lag^1^. Cumulative reports indicate that circadian rhythm disruptions are associated with the pathogenesis of many diseases, such as diabetes^2^, hypertension^3^ and chronic headaches^4^. Immune cell activities change with time of day^5^. The inflammatory pathologies are also associated with circadian rhythm disruption^6^. Published data indicate that the molecular clock, such as BMAL1, CLOCK, and REV-ERBα, controls fundamental immune responses^7^. Yet, factors that induce circadian rhythm disruption remain to be further understood.

Our previous studies showed that the regulatory B cell functions were disturbed in nurses engaging the day-night shift rotation^8^. The immune system in the body consists of immune regulatory cells [such as regulatory T cells (Treg), and regulatory B cells (Breg) or DEC205^+^ dendritic cells (decDC)] and immune regulatory mediators [such as interleukin (IL)-10 and transforming growth factor (TGF)-β]^9^. Published data indicate that dysfunction of the immune regulatory system is linked to the pathogenesis of many diseases, such as allergic diseases^9^ and autoimmune diseases^10^. Yet, the etiology of immune regulatory system dysfunction is not fully understood.

The association between circadian rhythm disruptions and immune diseases has been recognized^2–4^. Because immune responses in the body are tightly regulated by the immune regulatory system, the immune inflammatory conditions mirror the dysfunctional status of the immune regulatory system. It is known that probiotics have immune regulatory functions^11^. Probiotics are live micro-organisms distributing in the human body; when administered in adequate amounts, confer a health benefit on the host^12^. Ingestion of probiotics can alleviate immune disorders, such as inflammatory bowel disease^13^. As NS affects human immune activities^14^, we hypothesize that ingestion of probiotics may reconcile the effects of NS on compromising immune function. Therefore, in this study, we observed the effects of probiotics on regulating the decDC function of NS nurses. Blood samples were taken from nurses before and after NS. The decDC properties were evaluated by immune analyzing approaches.

## Results

### NS impairs decDC immune tolerogenic functions

**Figure 1:**
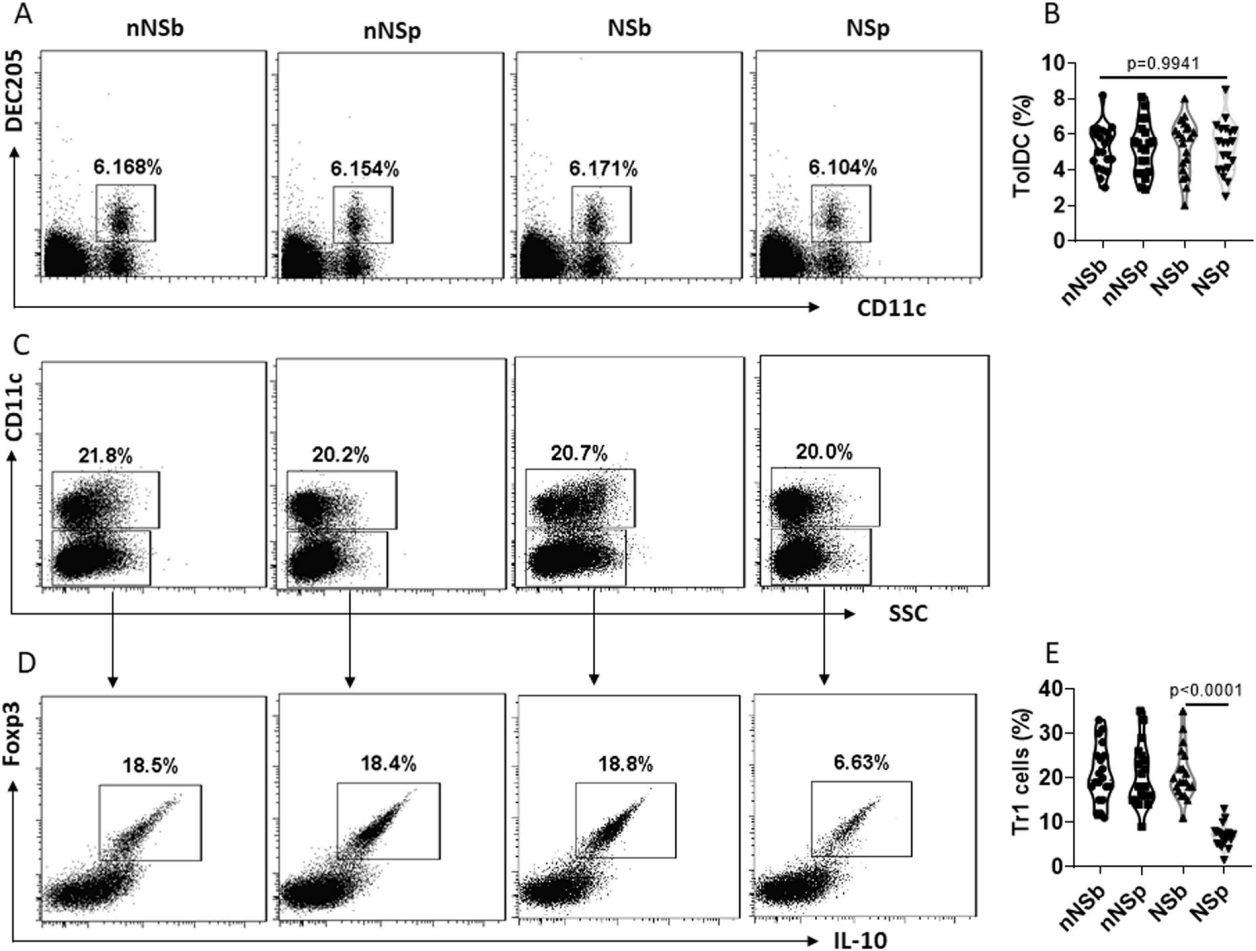
NS impairs decDC’s immune tolerogenic function. Blood samples were collected from nurses (n = 20) before (NSb) and after (NSp) NS, and collected from nurses (n = 20) engaged day shift only (non-NS, or nNS) at the same time points as of NS nurses. (**A,B**) Peripheral blood mononuclear cells (PBMCs) were isolated and analyzed by flow cytometry. The gated dot plots show frequency of decDCs (**A**). The violin plots show summarized frequency of decDCs (**B**). (**C–E**) decDCs were isolated from PBMCs. CD4^+^ CD25^−^ T cells were isolated from PBMCs collected from healthy subjects. DCs and T cells were cocultured at a ratio of 1:5 for 4 days in the presence of IL-2 (10 ng/ml), PMA (Phorbol 12-myristate 13-acetate; 0.1 µM) and ionomycin (100 ng/ml). (**C**) DCs were gated out first. (**D**) Gated dot plots show frequency of Tr1 cells. (**E**) Violin plots show summarized frequency of Tr1 cells. Each dot in panels (B,E) present data obtained from one sample. Statistics: ANOVA + Bonferroni test.

To assess the impact of NS on immune regulatory functions, peripheral blood samples were collected from nurses before and after NS. PBMCs were isolated from blood samples and analyzed by flow cytometry. The results showed that the frequency of decDCs was not significantly altered by NS (Fig. 1A,B). We then isolated decDCs from PBMCs. The decDCs were cocultured with CD4^+^ CD25^−^ T cells in the presence of PMA/ionomycin (non-specific cell activators) for 4 days. The cells were analyzed by flow cytometry. The results showed that NS significantly suppressed the immune tolerogenic functions of decDCs; markedly less type 1 regulatory T cells (Tr1) were induced by NSp decDCs (decDCs were collected from nurses after NS) than NSb decDCs (decDCs were collected from nurses before NS. Figure 1C–E). The results indicate that although NS does not alter the frequency of decDCs, it affects the decDC immune tolerogenic capacity.

### decDC IL-10 expression is down regulated by NS

**Figure 2:**
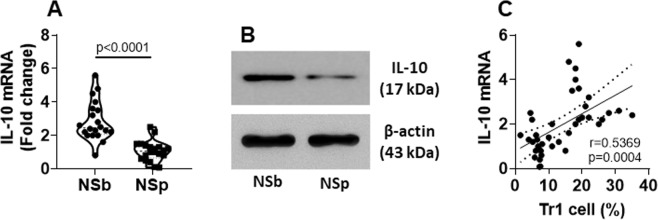
NS down regulates IL-10 expression in decDCs. decDCs were isolated from PBMCs (refer to Fig. 1 legend). (**A**) IL-10 mRNA levels in decDCs. (**B**) IL-10 proteins in decDCs. (**C**) A positive correlation between IL-10 mRNA in decDCs and the decDC-induced Tr1 cells. Each dot present data obtained from one sample. Protein samples were pooled per group. The data represent 3 independent experiments. Statistics: (**A**): *t* test. (**C**): Pearson correlation assay. (Full length gels are presented in the Supplemental Materials).

IL-10 is one of the major immune regulatory mediators of decDCs^15^. To test the effects of NS on IL-10 expression in decDCs, we isolated decDCs from PBMCs. The cells were analyzed by RT-qPCR and Western blotting. We observed that the IL-10 expression in decDCs was significantly reduced after NS (Fig. 2A,B). We also identified a positive correlation between the IL-10 expression and the Tr1 induction ability in decDCs (Fig. 2C). The results suggest that NS down regulates the IL-10 expression in decDCs, thus, impairs decDC immune regulatory capacity.

### NS increases HDAC11 expression in decDCs

**Figure 3:**
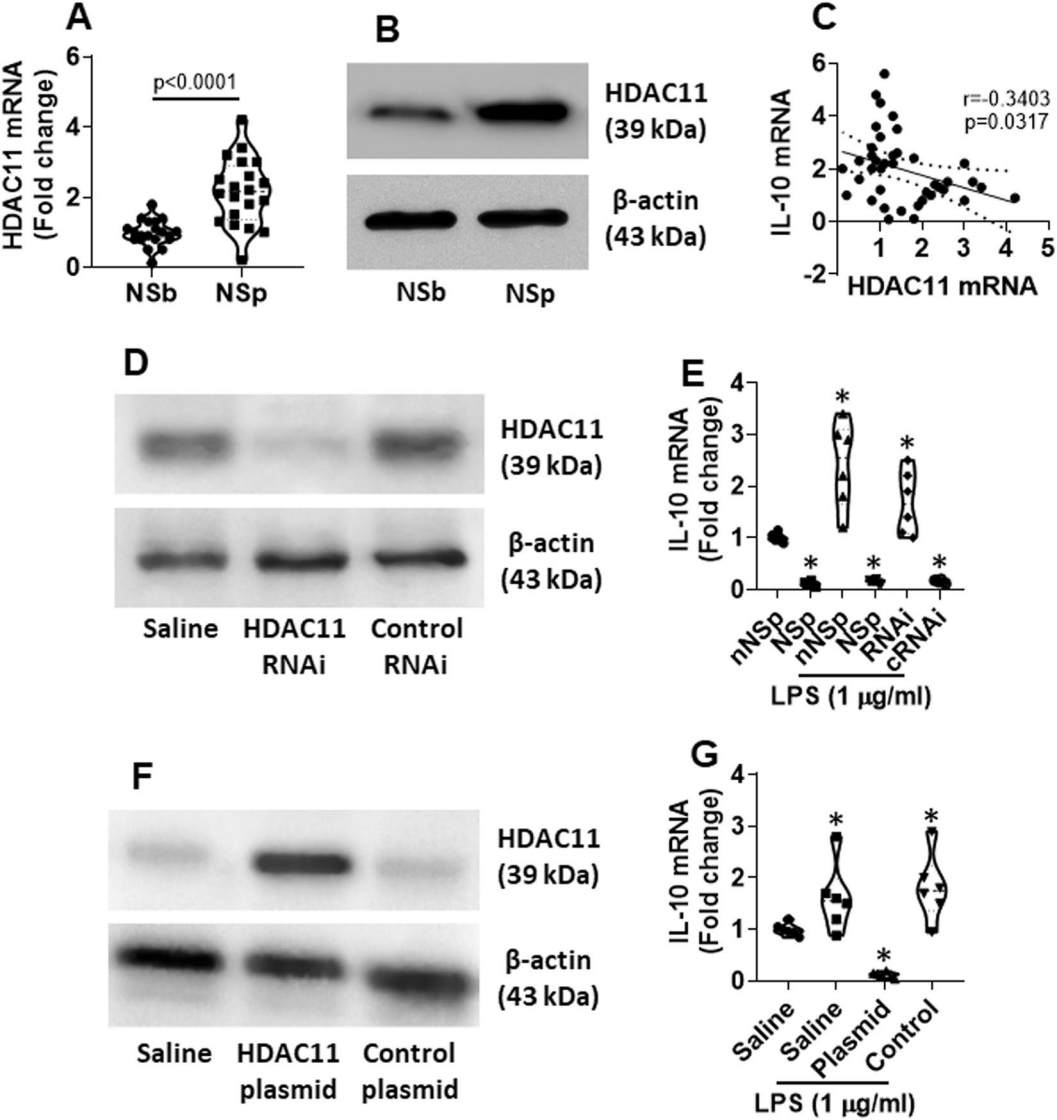
NS increases HDAC11 expression in decDCs. decDCs were isolated from PBMCs (refer to Fig. 1 legend). RNA and proteins were extracted from the decDCs and analyzed by RT-qPCR and Western blotting. (**A**) HDAC11 mRNA levels in decDCs. (**B**) HDAC11 protein levels in decDCs. **(C**) A negative correlation between HDAC11 mRNA and IL-10 mRNA in decDCs. (**D,E**) decDCs collected from NSp nurses were treated with HDAC11 RNAi (**D**) and exposed to LPS in the culture for 24 h. The violin plots (**E**) show IL-10 mRNA levels in decDCs. F-G, decDCs collected from nNSb nurses were transfected with HDAC11-expressing plasmids to make the cells over expression of HDAC11 (**F**). The cells were exposed to LPS in the culture for 24 h. The violin plots (**G**) show IL-10 mRNA levels in decDCs. Each dot present data obtained from each sample. Statistics: (**A**): *t* test. (**C**): Pearson correlation assay. (Full length gels are presented in the Supplemental Materials).

Published data indicate that HDAC11 can inhibit IL-10 expression^16^. Thus, we next assessed the possible association between NS and HDAC11 in decDCs. We found that the HDAC11 levels were markedly increased in decDCs after NS. The results indicate that NS can up regulate the activities of HDAC11 in decDCs (Fig. 3A,B). We further found that the levels of HDAC11 were negatively correlated with the IL-10 expression in decDCs in nurses after NS (Fig. 3C). Knock down of the HDAC11 gene expression restored the expression of IL-10 in decDCs collected from HSp nurses (Fig. 3D,E). Overexpression of HDAC11 in decDCs collected from non-NS nurses suppressed the IL-10 expression. The results indicate that NS suppresses decDC IL-10 expression through increasing HDAC11.

### Circadian protein Bmal1 is up regulated in decDCs by NS

**Figure 4:**
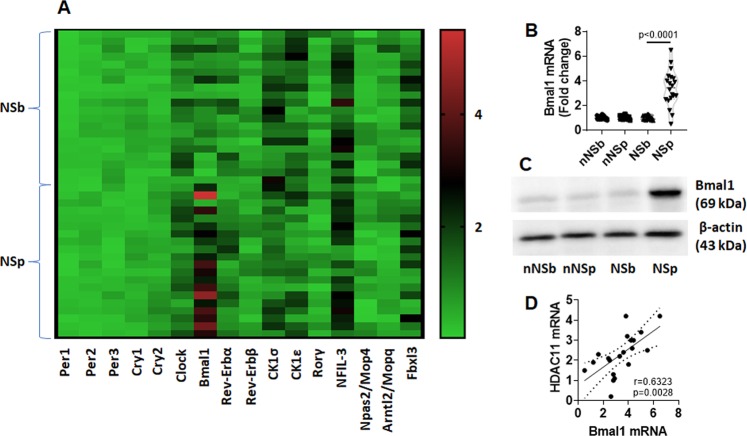
NS up regulates Bmal1 expression in decDCs. Blood samples were collected from 20 nurses before and after NS. (**A**) decDCs were isolated from the samples and analyzed by PCR array. Heat map shows circadian rhythm related gene activity in NSb and NSp samples. (**B**) Bmal1 mRNA levels in decDCs. (**C**) Bmal1 protein levels in decDCs. (**D**) positive correlation between Bmal1 mRNA and HDAC11 mRNA in decDCs. nNS: Non-NS nurse. NS: nurse engaging night shift. b: before NS. p: post NS. Each dot present data obtained from each sample. Statistics: (**B**): *t* test. (**D**): Pearson correlation assay. (Full length gels are presented in the Supplemental Materials).

It is known that NS can alter circadian protein expression^14^. To find a possible link between circadian proteins and the deregulation of decDC functions, we screened the circadian rhythm associated protein expression in decDCs of NS nurses. We found that, among the 20 circadian proteins, the activities of Brain muscle ARNT-like1 (Bmal1) were markedly higher in decDCs of NSp nurses than that in NSb samples and samples collected from non-NS nurses (Fig. 4A–C). The results demonstrate that NS increases the Bmal1 expression in decDCs of NS nurses. A positive correlation was also detected between the Bmal1 mRNA levels and the HDAC11 mRNA levels in decDCs (Fig. 4D), suggesting that Bmal1 may regulate the expression of HDAC11 in decDCs.

### Bmal1 increases HDAC11 expression in decDCs

**Figure 5:**
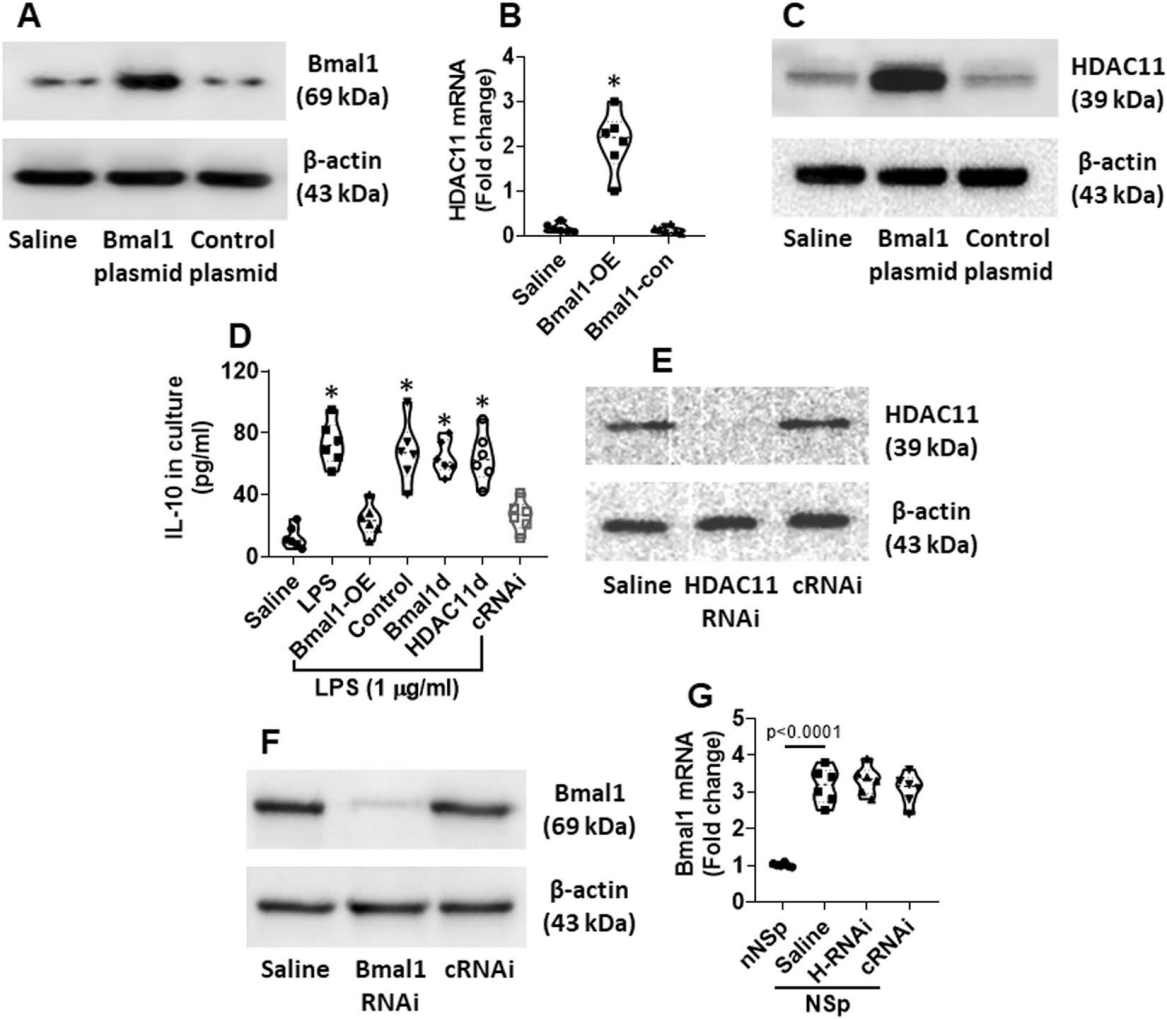
Bmal1 suppresses IL-10 expression in decDCs via up regulating HDAC11. decDCs were isolated from PBMCs (isolated from healthy subjects). To promote the expression of IL-10, decDCs were stimulated by LPS in the culture for 24 h. (**A**) decDCs were over expressed (OE) Bmal1 by transfecting with Bmal1-expressing plasmids. (**B,C**) IL-10 expression in decDCs. (**D**) IL-10 levels in culture supernatant. (**E**) Results of HDAC11 RNAi. cRNAi: Cells were treated with control RNAi. (**F**) RNAi results of Bmal1. (**G**) Bmal1 mRNA levels in decDCs. H-RNAi: HDAC11 RNAi. nNS: Non-NS nurses. Full length gels are presented in the Supplemental Materials. Statistics: ANOVA + Dunnett’s test.

To test the role of Bmal1 in regulating the HDAC11 expression, decDCs were isolated from healthy control (HC) subjects; the cells were transfected with Bmal1-expression plasmids to overexpress Bmal1 (Fig. 5A). This markedly increased the levels of HDAC11 in the decDCs (Fig. 5B,C), and the levels of IL-10 expression were down regulated subsequently (Fig. 5D). To verify the role of HDAC11 in the Bmal1-suppressed IL-10 expression in decDCs, HDAC11-deficient decDCs were prepared (Fig. 5E) and transfected with Bmal1-expression plasmids. The depletion of HDAC11 indeed abolished the Bmal1-suppressed IL-10 expression in decDCs (Fig. 5D). In addition, depletion of Bmal1 (Fig. 5F) also increased the IL-10 expression (Fig. 5D). The results demonstrate that Bmal1 can suppress the IL-10 expression in decDCs through up regulating the HDAC11 expression. On the other hand, the depletion of HDAC11 did not affect the Bmal1 expression in decDCs (Fig. 5G), indicating that Bmal1 is in the upper stream of the signal transduction pathway.

### Probiotics prevent NS-induced decDC dysfunction

**Figure 6:**
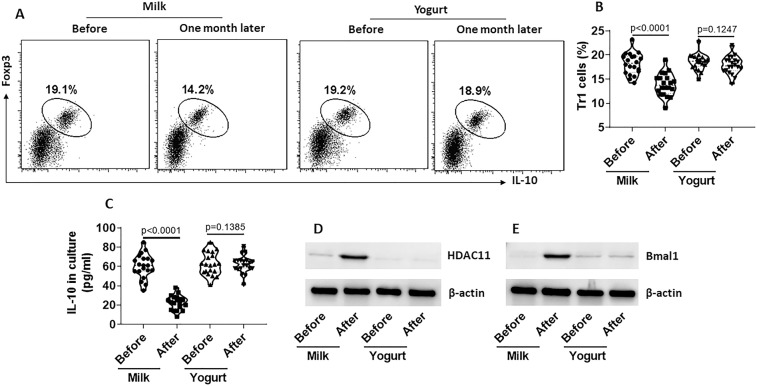
Yogurt ingestion prevents NS-induced decDC dysfunction. Nurses took milk (100 ml) (n = 20) or yogurt (100 g) (n = 20) during NS for one month. Blood samples were collected from each nurse before and after the ingestion of milk or yogurt. PBMCs were isolated from the samples; decDCs were further isolated from PBMCs. CD4^+^ CD25^−^ T cells were isolated from blood samples collected from healthy subjects. DCs and T cells were cocultured at a ratio of 1:5 for 4 days in the presence of IL-2 (10 ng/ml), PMA (0.1 µM) and ionomycin (100 ng/ml). (**A**) cells were analyzed by flow cytometry. DCs were gated out first (not shown). Frequency of Tr1 cells in remained T cells was determined. The gated dot plots show frequency of Tr1 cells. (**B**) Summarized data of panel A. (**C**) IL-10 levels in the culture supernatant (by ELISA). (**D,E**) decDCs were analyzed by Western blotting. The immunoblots show protein levels of HDAC11 (**D**) and Bmal1 (**E**) in decDCs. Each dot of (**B,C**) present data obtained from one sample. Statistics: ANOVA + Bonferroni test.

Previous reports indicate that probiotics improve immune functions^17^. Thus, we tested the effects of probiotic-containing yogurt ingestion on counteracting the NS-induced decDC dysfunction. Blood samples were collected from nurses ingested 100 ml milk or 100 g yogurt (containing about 10^10^ C. butyricum) during NS. decDCs were isolated from the blood samples and analyzed for the tolerogenic properties. We found that yogurt ingestion for one month in NS efficiently maintained the capacity of decDCs in the induction of Tr1 cells (Fig. 6A,B) and the production of IL-10 (Fig. 6C), and kept the levels of HDAC11 and Bmal1 not increased by NS (Fig. 6D,E). The results demonstrate that ingestion of probiotic containing yogurt protects decDCs of NS nurses from impairing the immune tolerogenic functions.

## Discussion

The present data show that NS can down regulate decDCs’ immune tolerogenic capacity by inhibiting IL-10 expression. We observed that NS increased the expression of Bmal1 in decDCs; Bmal1 increased the expression of HDAC11; the latter suppressed IL-10 expression in decDCs. Ingestion of probiotic containing yogurt prevented the NS-induced decDC dysfunction.

The day-night shift rotation occurs frequently in people engaging specific occupation. NS is a regular shift in hospitals. Published data reveal that NS can alter the circadian rhythm^18^. There are several diseases occur in people engaging day-night shift rotation more often than people with other careers. For example, Night-shift work is one of the causative factors associating with poorer glycaemic control in type 2 diabetes patients^2^. Engaging day-night shift rotation for one year has risk of incident hypertension^3^. Nurses with day-night shift rotation reported higher prevalence of frequent or chronic headaches^4^. The present data provide further evidence that NS may contribute to compromising human health.

The data show that, after NS, decDC immune tolerogenic efficacy is compromised. decDCs are an important cell fraction in immune regulatory activities in the body. By releasing immune regulatory mediators, decDCs induce regulatory T cells^19^. Previous reports indicate that DCs are dysfunctional in subjects with food allergy^20^. In steroid resistant asthma patients, the myeloid DC phenotype is increased^21^. Yet, the etiology of decDC dysfunction is less understood currently. The present data show that the immune tolerogenic capacity of decDCs are impaired in subjects after NS, suggesting that NS may be one of the causative factors inducing decDC dysfunction.

We found that the IL-10 expression in decDCs was decreased after NS. IL-10 is a canonical immune regulatory mediator in decDCs^22^. IL-10 deficiency can result in immune inflammation^23^ and cannot generate immune tolerance^24^. In this study, we observed that the NS-induced Bmal1 was negatively correlated with IL-10 expression in decDCs. Bmal1 is one of the circadian rhythm proteins. Expression of Bmal1 can be regulated by circadian rhythm disruptions. Sleep deprivation is one of the factors increasing Bmal1 expression^25^. Our data show that NS up regulated Bmal1 expression in decDCs. As both Bmal1 and IL-10 expression were altered by NS, there may be a link between these two factors in decDCs.

We also found that the levels of HDAC11 were increased in decDCs after NS. HDAC11 is a member of the HDAC family. HDAC family plays an important role in regulating gene transcription under physiological conditions^26^. Dysregulation of HDAC is associated with the pathogenesis of many diseases. Inhibition of HDAC has been employed in the treatment of many diseases such as cancer^27^, diabetes^28^ and allergic diseases^29^. Our data are consistent with those previous reports. We found that HDAC11 was negatively correlated with the levels of IL-10 in decDCs. Furthermore, the data indicate that NS can increase HDAC11 levels in decDCs, suggesting that NS is one of the factors regulating the activities of HDAC11.

The present data show that NS increases HDAC11 and then impairs decDCs’ tolerogenic capacity. This is a harmful event to the immune homeostasis of the body. However, there are not many remedies available to prevent impairing DC’s tolerogenic property or restore the impaired DC’s tolerogenic capacity. The present results contribute to this area by showing that ingestion of probiotic containing yogurt can prevent the NS-induced decDC dysfunction. Probiotics have been used as a supplement to therapeutics for immune disorders^17^. Our data are in line with previous studies by showing that ingestion of probiotics can prevent the effects of NS on compromising immune tolerant activities in the body.

In summary, the present data show that NS can compromise decDCs’ tolerogenic capacity by increasing Bmal1 and HDAC11, and suppressing IL-10 expression in decDCs, which can be prevented by ingestion of probiotic containing yogurt.

## Materials and Methods

### Reagents

HDAC11 (sc-106896) and Bmal1 (sc-38165) shRNA kit and antibodies of IL-10 (sc-32815), HDAC11 (sc-390737) and Bmal1 (sc-365645) were purchased from Santa Cruz Biotech (Santa Cruz, CA). Fluorescence labeled antibodies, including PE-DEC205, FITC-CD11c, PE-Foxp3 and Alexa Fluor647-IL-10, were purchased from BD Biosciences (Franklin Lakes, NJ). The IL-10 ELISA kit was purchased from R&D Systems (Minneapolis, MN). QuantiTect Reverse Transcription Kit and SYBR Green qPCR Master Mix were purchased from Qiagen (Germantown, MD). Materials and reagents for RT-qPCR and Western blotting were purchased from Invitrogen (Carlsbad, CA).

### Ethic statement

The experimental procedures were approved by the Human Ethics Committee at Shanxi Medical University. All methods were performed in accordance with the relevant guidelines and regulations. A written informed consent was obtained from each human subject.

### Human subjects

Nurses engaging the day-night rotation shift for 1–4 years were randomly recruited into this study. The demographic data are presented in Table 1. Subjects with any of the following conditions were excluded from this study, including allergic diseases, autoimmune diseases, severe organ diseases, cancer, under treatment with corticosteroids or other immune tolerogenic agents for any reasons.

**Table 1:** Demographic data.

	NS nurses	nNS nurses
Number	20	20
Age (years)	35.5 ± 2.5	33.8 ± 3.1
Gender	Female	Female
Years of nursing (years)	2.5 ± 1.1	2.6 ± 1.2
Serum total IgE	<0.35 IU/ml	<0.35 IU/ml
Regular menstrual	20 (100%)	20 (100%)
Blood glucose (mmol/L)	4.55 ± 1.56	4.61 ± 1.85
Second job nurse	0	0
Sleep disorder nurse	0	0
Mood disorder nurse	0	0

### Preparation of peripheral blood mononuclear cells (PBMC)

Blood samples were collected from nurses through ulnar vein puncture before and after each NS shift (the NS shift time was 12 h) at 20 ml per case. To avoid the blood drawing stress, nurses were explained the study purposes before the experiments. PBMCs were isolated from blood samples by Percoll gradient density centrifugation.

### Cell culture

Cells were cultured in RPMI1640 medium. The medium was supplemented with fetal calf serum (10%), glutamine (2 mM), penicillin (100 U/ml) and streptomycin (0.1 mg/ml). The medium was changed in 2–3 days. Cell viability was greater than 99% as assessed by Trypan blue exclusion assay.

### Immune cell isolation

DCs, B cells and CD4^+^ T cells were isolated from PBMCs by flow cytometry cell sorting. The cells were labeled by incubating with fluorescence-conjugated antibodies and sorted with a flow cytometer. If the purity of isolated cells did not reach 95%, the cell sorting was repeated.

### Flow cytometry

Cells were collected from relevant experiments. In the surface staining, cells were stained with fluorescence labeled antibodies of interest or isotype IgG for 30 min at 4 °C. In the case of intracellular staining, cells were fixated with 1% paraformaldehyde (containing 0.1% Triton X-100 to increase cell membrane permeability) for 1 h. Then, cells were stained with fluorescence labeled antibodies of interest or isotype IgG for 30 min at 4 °C. After washing with PBS, cells were analyzed with a flow cytometer (FACSCanto II; BD Bioscience). Data were analyzed using a software package, FlowJo (TreeStar Inc., Ashland, OR) Data obtained from isotype IgG staining were used as a gating reference.

### Assessment of decDC tolerogenic functions

Naive CD4^+^ CD25^−^ T cells were isolated from blood samples collected from healthy subjects; decDCs were isolated from blood samples collected from nurses before and after NS. CD4^+^ CD25^−^ T cells were cultured with decDCs at a ratio of 5:1 in the presence of PMA (50 ng/ml) and ionomycin (100 ng/ml) for 4 days. Then, the cells were analyzed by flow cytometry. The frequency of IL-10^+^ Foxp3^+^ iTr1 cells was determined and used as an indicator of decDC immune tolerogenic functions.

### Real-time quantitative RT-PCR (RT-qPCR)

Total RNA was extracted from cells collected from relevant experiments and converted to cDNA with a reverse transcription kit following the manufacturer’s instructions. The samples were amplified in a qPCR device with the SYBR Green Master Mix in the presence of relevant primers (Table 2). The results were processed with the 2^−∆∆Ct^ method and presented as fold change against controls.

**Table 2:** Primers used in the study.

	Forward	Reverse
IL-10	gccaagccttgtctgagatg	aagaaatcgatgacagcgcc
HDAC11	tgtctacaaccgccacatct	cggtgcctgcattgtatacc
Bmal1	ccctgggccatctcgattat	tcatccagccccatctttgt

### Western blotting

Total proteins were extracted from cells collected from relevant experiments, fractioned by SDS-PAGA and transferred onto a PVDF membrane. After blocking with 5% skim milk for 30 min, the membrane was incubated with the primary antibodies (diluted 1:200–300) of interest overnight at 4 °C, washed with TBST (Tris-buffered saline containing 0.1% Tween 20) 3 times, incubated with peroxidase-labeled secondary antibodies for 2 h and washed with TBST 3 times. Immunoblots on the membrane were developed by the enhanced chemiluminescence and photographed in an imaging device (UVP imaging; Cambridge, UK).

### Over expression of Bmal1 or HDAC11 in decDCs

decDCs were isolated from healthy subjects and transfected with Bmal1-expressing or HDAC11-expressing plasmids or control plasmids (provided by Sangon Bioteck, Shanghai, China) following the manufacturer’s instructions. The effects of Bmal1 or HDAC11 overexpression was assessed 48 h later by Western blotting.

### Depletion of HDAC11 in decDCs

decDCs were treated with HDAC11 RNAi reagents following the manufacturer’s instructions. The effects of HDAC11 depletion were assessed by Western blotting 48 h later.

### Ingestion of yogurt or milk during night shift

NS nurses were randomly divided into two groups. One group was provided with yogurt (containing *Clostridium butyricum*; produced by KeEn Food Ltc., Taiyuan, China. 100 g/NS), another group was provided with milk (produced by KeEn Food Ltc., Taiyuan, China. 100 ml/NS). One month later, blood samples were collected from each nurse as described above.

### Statistics

Data are presented as mean ± SEM. The difference between two groups was determined by Student t test. ANOVA followed by Dunnett’s test or Bonferroni test was performed for multiple comparisons. The correlation between two data sets were determined by Pearson correlation assay. P < 0.05 was considered statistical significance.

## Supplementary information

supplemental materials
