# Supplementary material for: Probiotics ingestion prevents HDAC11-induced DEC205+ dendritic cell dysfunction in night shift nurses
Source: Sci Rep. 2019 Nov 29;9:18002. doi: 10.1038/s41598-019-54558-4 (PMC6884592; doi:10.1038/s41598-019-54558-4)

Supplemental Materials

Probiotics ingestion prevents HDAC11-induced DEC205+ dendritic cell dysfunction in night shift nurses

Running title: Probiotics prevent decDC dysfunction

Hui Yang[1], Jing Yang[1], Hui Cheng[1], Huili Cao[1], Shan Tang[1], Qiaohong Wang[1], Juan Zhao[1], Baohua Li[1], Yongxia Ding[1], Chang Ma[2]

1, Department of Nursing, First Hospital of Shanxi Medical University, Taiyuan, China.
2, Department of Respirology, Second Affiliated Hospital, Harbin Medical University, Harbin, China.

Corresponding author: Hui Yang. Department of Nursing, First Hospital of Shanxi Medical University, Taiyuan 030001, China. Email: qyhui2020@163.com. Tel: 863514044111. Fax: 863514044112.

Fig. 2B

IL-10 β-actin

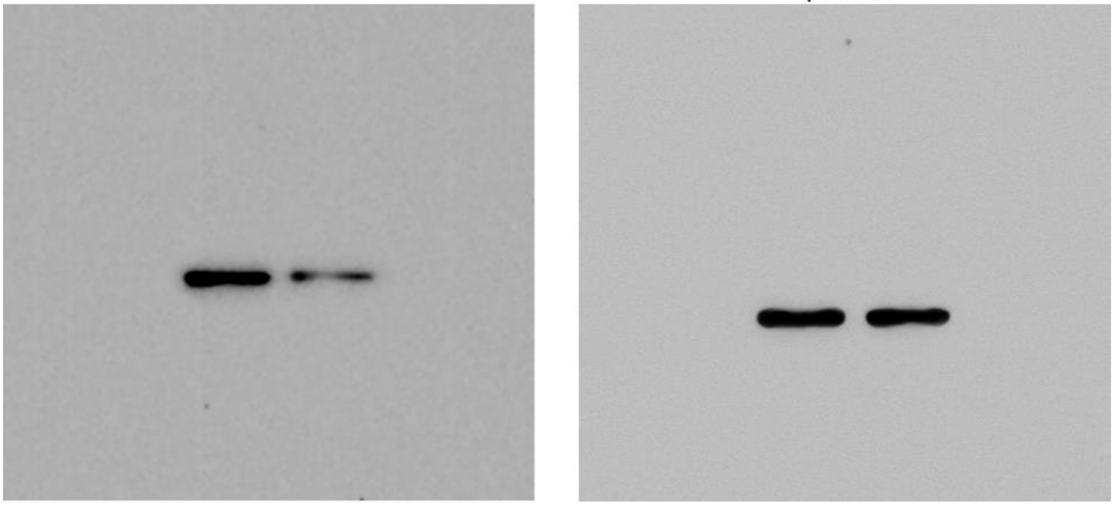

Fig. 3B

HDAC11 β-actin

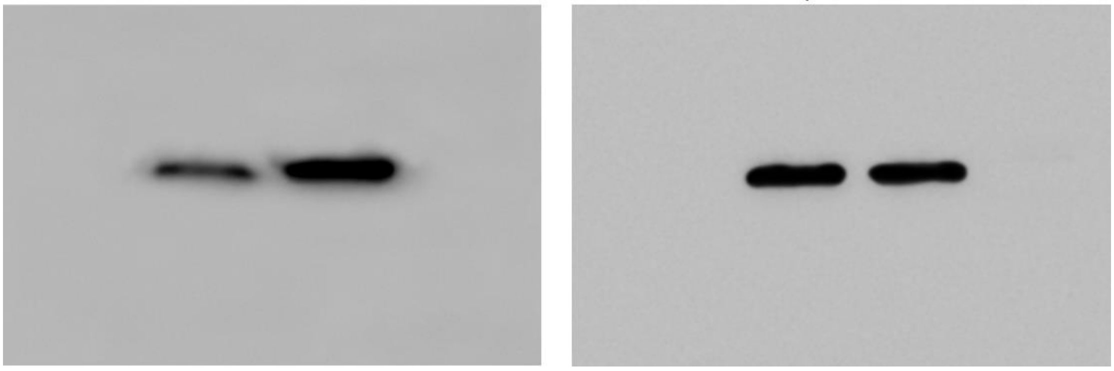

Fig. 3D

HDAC11 **β-actin**

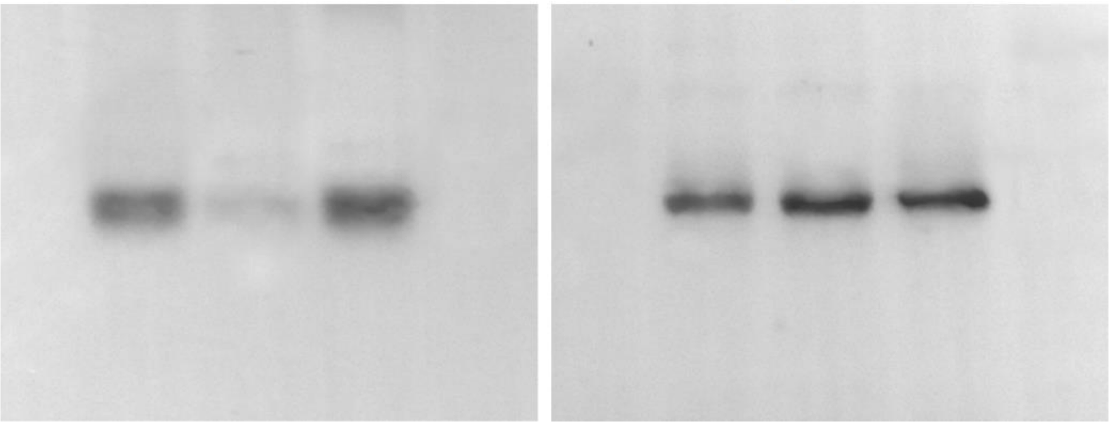

Fig. 3F

HDAC11 β-actin

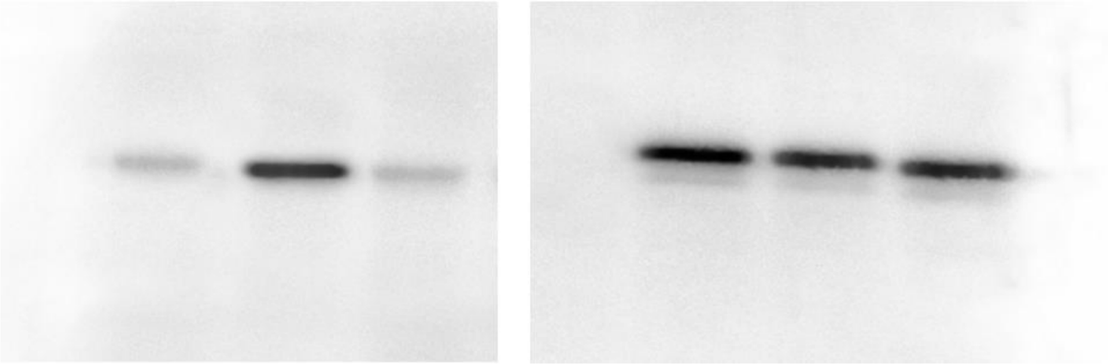

Fig. 4C

Bmal1 **β-actin**

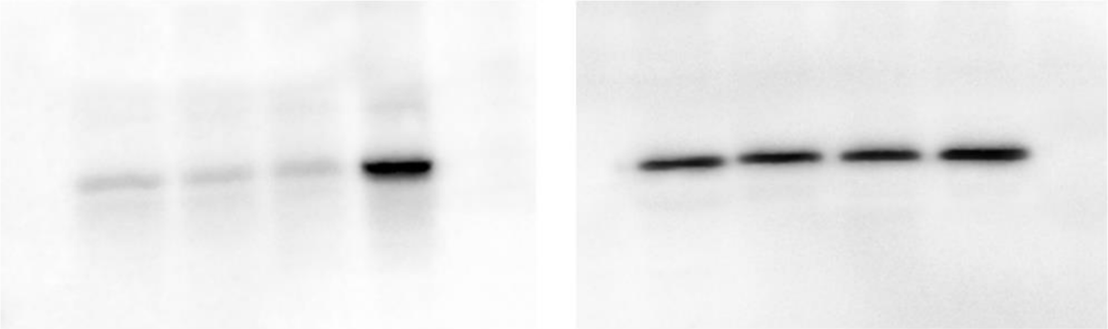

Fig.5c

HDAC11 β-actin

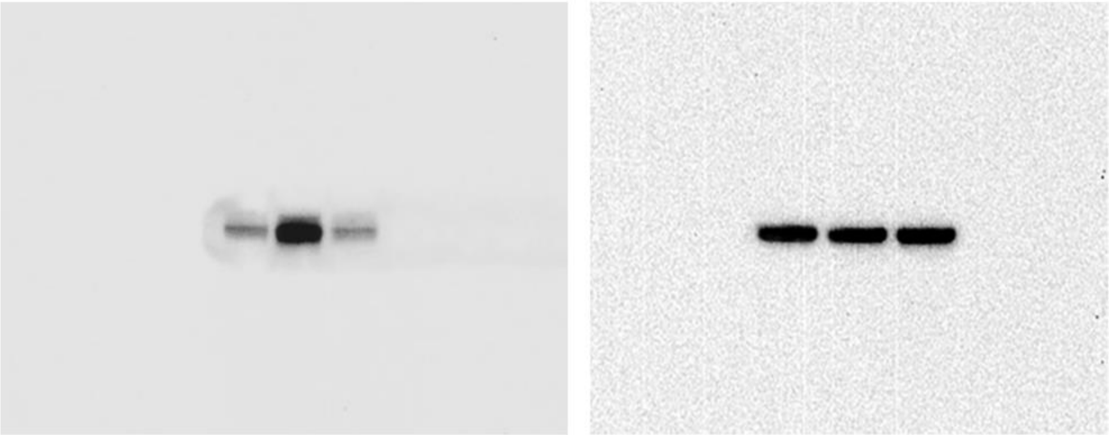

Fig. 5D

Bmal1 **β-actin**

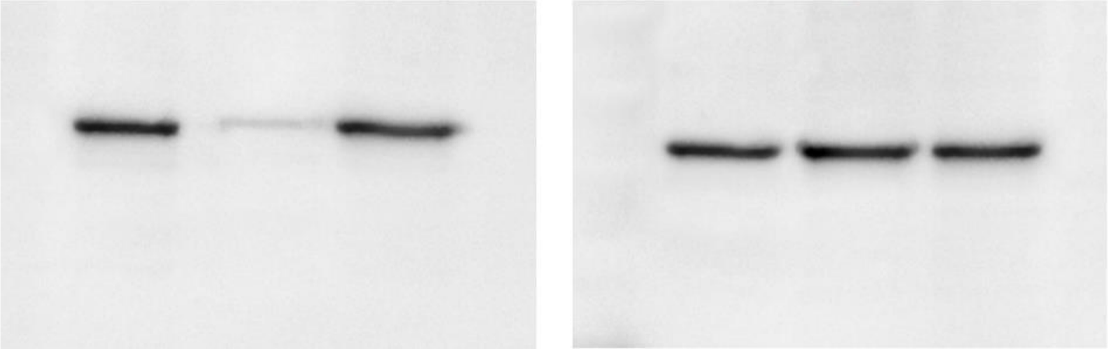

Fig. 5E

HDAC11 β-actin

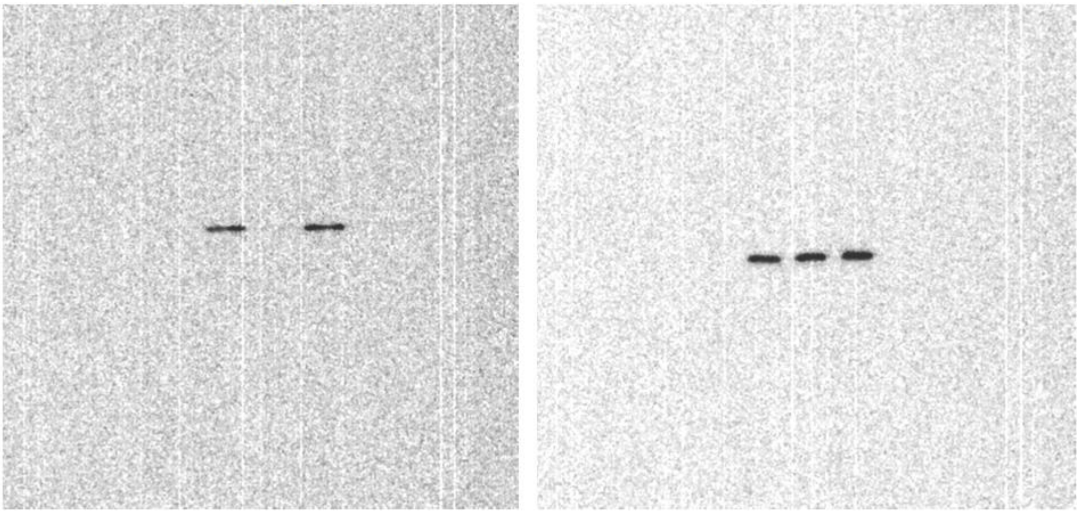

Fig. 6D

HDAC11 **β-actin**

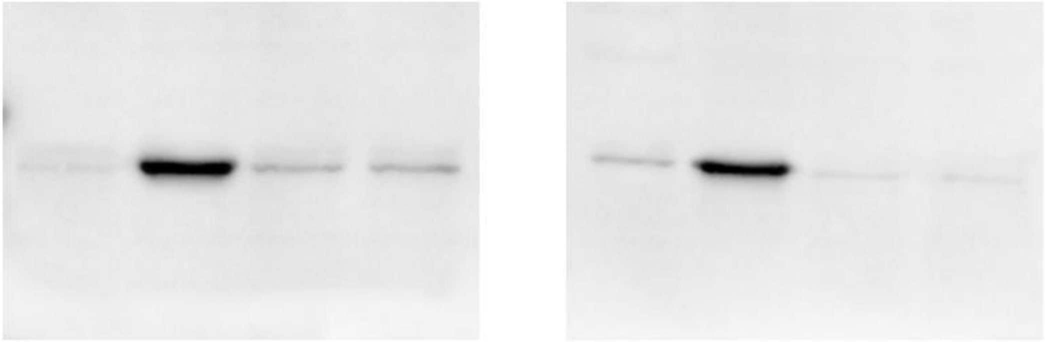

Fig. 6E

Bmal1 **β-actin**

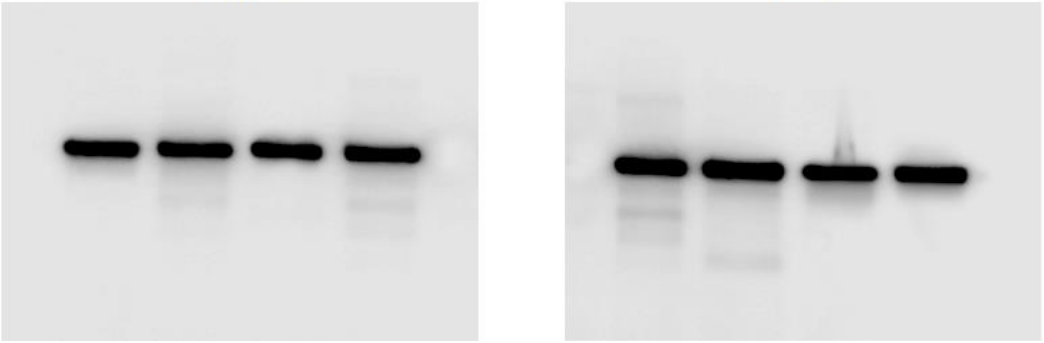

Supplement: Supplementary file 1 — supplemental materials [file 41598_2019_54558_MOESM1_ESM.pdf]
